# Post-surgical hemodynamics in aortic valve bypass (AVB) patients evaluated with phase contrast magnetic resonance (PCMR)

**DOI:** 10.1186/1532-429X-14-S1-P95

**Published:** 2012-02-01

**Authors:** Adrian Lam, Stephanie Clement-Guinaudeau, Muralidhar Padala, Vinod Thourani, John N Oshinski

**Affiliations:** 1 Georgia Institute of Technology, Atlanta, GA, USA; 2 Emory University, Atlanta, GA, USA

## Background

**Figure 1:**
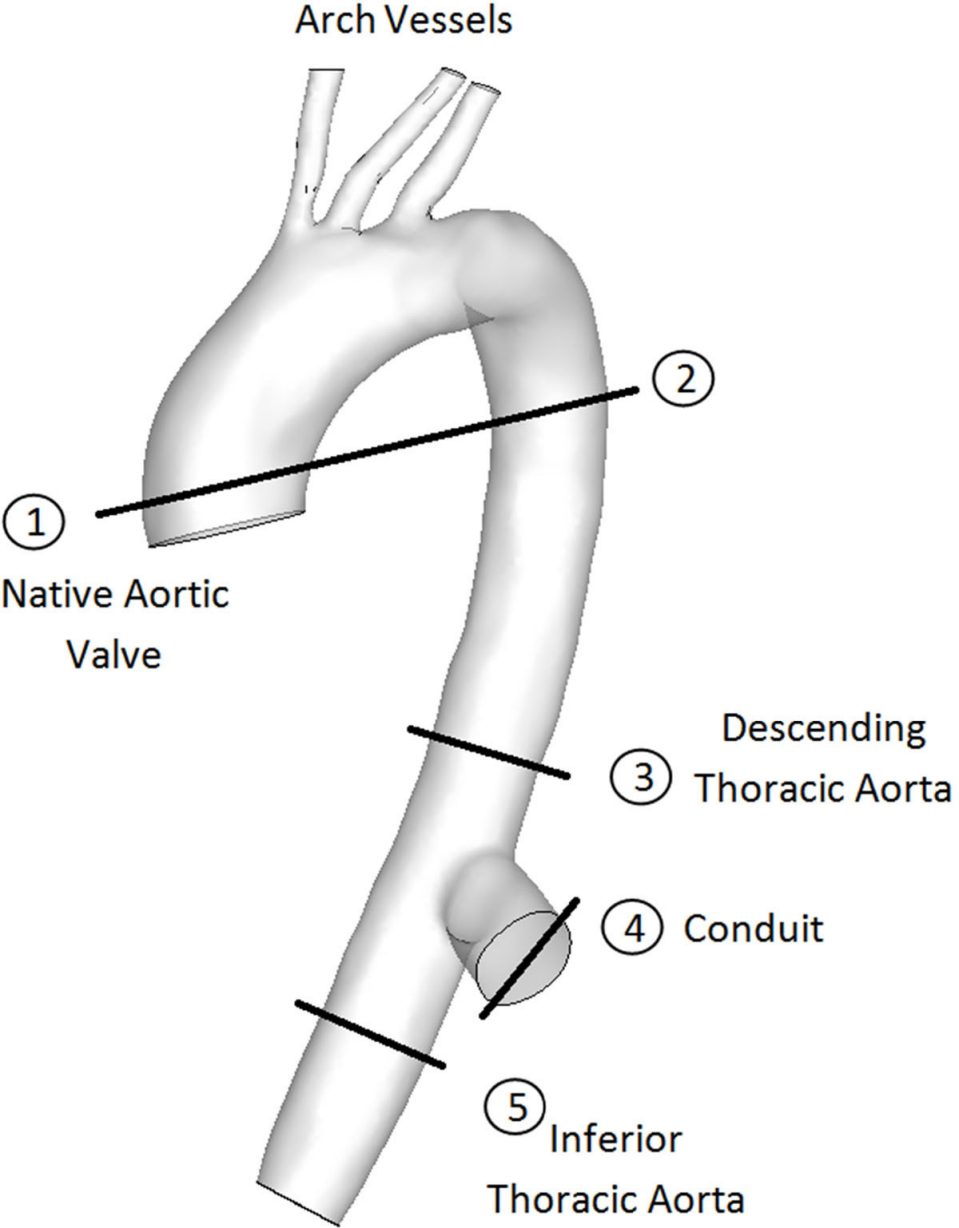
Aortic valve bypass surgery. Lines indicate PCMR image acquisition locations.

Many high-risk patients with severe aortic stenosis cannot undergo valve replacement surgery due to calcification of the ascending aorta. Aortic valve bypass (AVB) surgery uses a conduit and prosthetic valve placed transapically into the left ventricle to divert flow from the apex of the heart through the conduit and prosthetic valve to the descending thoracic aorta to improve cardiac output, Fig 1. The hemodynamics resulting from AVB are not well understood. Specifically, there appears to be significant patient-to-patient variability in the amount of retrograde blood flow in the descending thoracic aorta (location 3, flow from conduit to arch vessels). The objective of this study is to use phase contrast magnetic resonance (PCMR) to examine the hemodynamics in AVB patients and determine the relationship between pre-surgery native aortic valve pressure gradient and post-surgery retrograde blood flow in the thoracic aorta.

## Methods

20 patients were included in this study. Mean pressure gradient across the aortic valve pre-surgery (ΔP) was determined with Doppler ultrasound using the modified Bernoulli equation. Patients underwent an MRI post-surgery, and PCMR images were acquired along the aorta and in the graft conduit at 5 locations, Fig 1.

## Results

**Figure 2:**
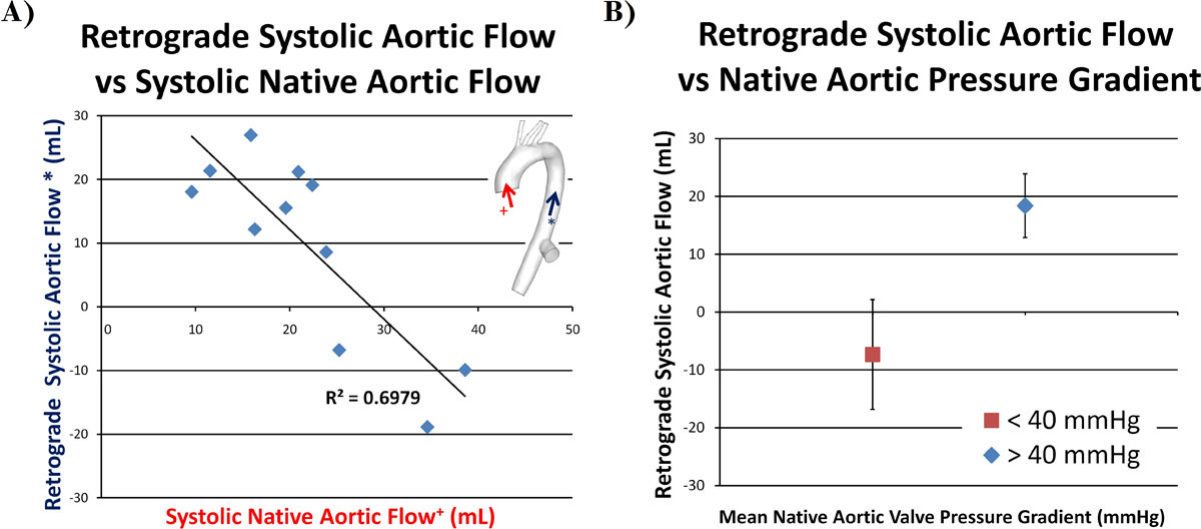
A) Retrograde systolic aortic blood flow in the descending aorta is inversely correlated to systolic native aortic flow. B) Patients with pre-surgery aortic valve pressure gradients greater than 40 mmHg will likely have retrograde aortic blood flow.

Flow curves generated from PCMR showed large patient-to-patient variations in the flow patterns at the descending thoracic aorta (location 3). Patients were categorized into three groups: 1) patients with mono-phasic *retrograde* flow over the cardiac cycle in the descending thoracic aorta (10/20), 2) patients with mono-phasic *antegrade* flow over the cardiac cycle in the descending thoracic aorta (5/20) and 3) patients with multi-phasic (mixed antegrade and retrograde) flow (5/20). The amount of retrograde flow in the descending thoracic aorta (location 3) was inversely correlated to blood flow from the native aorta (location 1), Fig 2a. This indicates that despite variations in the direction or source of the flow, the volume of blood flow to the arch vessels remains relatively constant among patients. Additionally, patients were divided into two groups: those with pre-surgery ΔP<40 mmHg or those with ΔP>40mmHg, Fig 2b. Patients with ΔP<40 had significantly less retrograde flow than those with ΔP>40 mmHg (p<0.05) and flow in those patients was not significantly different from zero (p<0.05).

## Conclusions

While much patient-to-patient variability exists in the flow patterns post AVB surgery, retrograde blood flow in the descending thoracic aorta is related to pre-surgery aortic valve gradient. This relationship may help with future patient selection, as patients with pre-surgery native aortic valve pressure gradient >40 mmHg will most likely have retrograde flow supplying the arch vessels.

## Funding

This project was funded by the NIH T32 Training Grant.

